# Trading green backs for green crabs: evaluating the commercial shellfish harvest at risk from European green crab invasion

**DOI:** 10.12688/f1000research.2-66.v3

**Published:** 2014-10-16

**Authors:** Megan E Mach, Kai MA Chan

**Affiliations:** 1 Institute for Resources, Environment and Sustainability, Resource Management and Environmental Sustainability, University of British Columbia, Vancouver, V6T 1Z4, Canada; 2 Current address: Center for Ocean Solutions, Monterey, CA, 93940, USA

## Abstract

Nonnative species pose a threat to native biodiversity and can have immense impacts on biological communities, altering the function of ecosystems. How much value is at risk from high-impact invasive species, and which parameters determine variation in that value, constitutes critical knowledge for directing both management and research, but it is rarely available. We evaluated the value of the commercial shellfish harvest that is at risk in nearshore ecosystems of Puget Sound, Washington State, USA, from the invasive European green crab, *Carcinus maenas*. We assessed this value using a simple static ecological model combined with an economic model using data from Puget Sound’s shellfish harvest and revenue and the relationship between *C. maenas *abundance and the consumption rate of shellfish. The model incorporates a range in *C. maenas* diet preference, calories consumed per year, and crab densities. *C. maenas* is likely to prey on commercially harvested hardshell clams, oysters, and mussels, which would likely reduce additional revenue from processing and distribution, and the number of jobs associated with these fisheries.

The model results suggest possible revenue losses of these shellfish ranging from $1.03-23.8 million USD year ^-1^ (2.8-64% losses), with additional processing and distribution losses up to $17.6 million USD and 442 job positions each year associated with a range of plausible parameter values. The broad range of values reflects the uncertainty in key factors underlying impacts, factors that are highly variable across invaded regions and so not knowable *a priori*. However, future research evaluating species invasions can reduce the uncertainty of impacts by characterizing several key parameters: density of individuals, number of arrivals, predation and competition interactions, and economic impacts. This study therefore provides direction for research to inform more accurate estimates of value-at-risk, and suggests substantial motivation for strong measures to prevent, monitor, and manage the possible invasion of *C. maenas*.

## Introduction

In coastal ecosystems, preventing and mitigating the spread and impacts of nonnative species has become a global priority ^1, 2^. While many nonnative species have little to no measurable impact on their invaded regions, a few have caused great economic and ecosystem harm ^3, 4^. The impacts from these few invasive species can affect ecosystem function and thereby reduce the benefits that ecosystems provide for people ^5– 9^. In the USA, the cost of invasive species impacts has been estimated at over $120 billion USD per year ^3, 10^. One example, the Floating Bell jellyfish, *Phyllorhiza punctate*, has been estimated to have cost $10 million USD in lost shrimp landings due to clogged nets (reviewed in Williams, S. L. & Grosholz (2008) ^11^). With limited funds to manage and research coastal ecosystems, calculating the value-at-risk (the losses that might accompany the establishment of a high-impact invasive species prior to introduction) for areas not yet invaded may justify the allocation of resources to prevent, mitigate, or further understand invasive impacts ^12, 13^.

The estimation of value-at-risk from invasive species should be distinguished clearly from a purpose of prediction. Rather than a statement of what is expected to occur, value-at-risk can provide decision-makers with a sense of what might plausibly be lost to invasion without prevention or mitigation. Whereas in finance, value-at-risk is often a monetary quantity subject to loss with a given probability ^14^, the concept might also be useful in settings where data limitations restrict the explicit assignment of probabilities. For example, a decision-maker faced with the decision whether to fund action to prevent biological invasion, or to put in place mechanisms to mitigate losses should invasion occur, might only wish to know what value—in revenues, jobs, etc.—might plausibly be subject to loss, based on our current understanding and its limitations. For such decisions, which are faced everyday, waiting for better data or a more sophisticated model may not be an option. Just as this quantity of value-at-risk can motivate management action, it can also motivate research to improve estimates of value-at-risk, and perhaps even enable prediction.

Economic value-at-risk can be seen as a product of two components: ecological (plausible ecological changes that might result from the introduction of a known invader) and economic (the economic costs that might be associated with the above ecological changes or resulting mitigation). Ecological consequences have been assessed mainly as projected post-invasion impacts or pre-invasion estimated ecosystem changes ^15– 17^. For example, ecological impact estimates of the European green crab, *Carcinus maenas*, suggest a future loss of valuable habitats and native species abundance in invaded areas, while larvae of the green crab may also provide food resources to migrating salmon in the northeast Pacific ^15^.

The economic consequences of invasive species can be estimated through damages to resources ^3, 4, 18^. However, many studies that evaluate economic costs do so with considerable simplification of important ecological processes, potentially misrepresenting costs ^19– 21^. For example, damages have been measured as reduced fishery values since time of invasion ^22, 23^, but these estimates do not consider other potential causal factors or variation in key traits of the native or invading species, such as varying invasion densities, predation rates or varying spatial distribution of native species. Economic valuations can better represent impacts of nonnative species if they incorporate specific information on both the native and invasive populations and their potential interactions ^20^.

There are great uncertainties in predicting impacts of species invasions ^24, 25^, in part because impacts vary across space and time and are otherwise context-dependent ^26– 30^. To be useful, evaluations made before invasions occur should incorporate economic costs and uncertainties associated with the invasion’s possible ecological consequences ^21, 31^. If value-at-risk estimates are structured to enable explicit assessment of the uncertainties associated with key parameters, even a coarse understanding of potential impacts can yield useful assessments. Ecological-economic models offer the opportunity to estimate economic changes that include these uncertainties ^19^. Though the value-at-risk of predicted invasions has not frequently been calculated, preemptive management strategies that incorporate ecological modeling could have considerable economic benefits ^13, 19^. For example, if early preventative efforts had been funded to prevent invasion of the rusty crayfish, *Orconectes rusticus*, $6 million USD in fisheries harvest revenue during the 30 years since invasion may have been protected ^13^. These calculations serve a critically important purpose of aligning future research and management ^25^.

Here we assessed the value-at-risk for commercial shellfish harvest in nearshore ecosystems of Puget Sound, Washington State, USA, from the green crab *C. maenas*, a species already regarded as a threat to ecosystems in Puget Sound ^32, 33^. To assess value-at-risk, we combined an ecological model of risk to shellfish harvest biomass by *C. maenas* with an economic model demonstrating risk to current shellfish harvest revenue. The secondary economic effects were predicted using benefit transfer from a similar neighbouring region, British Columbia, Canada (BC). Furthermore, we characterized the extent to which variation in key parameters influenced the resulting value-at-risk.

## Methods

### Organism and study site: *Carcinus maenas* as a threat to Puget Sound

Nonnative species with broad physiological tolerances and diverse diets, such as *Carcinus maenas*, are well suited to take advantage of available resources and out-compete native species ^34^. *C. maenas* is a generalist predator that, in one study, was found to consume species from at least 104 families and 158 genera within 14 animal and 5 plant and protozoan phyla ^35^, though it most commonly feeds on bivalves ^36, 37^. In feeding trials, *C. maenas* was able to feed more generally and consume a greater biomass than several native northeast Pacific *Cancer* crab species ^35^. Green crab have destroyed artificial shellfish beds and consumed juvenile bivalves and *Cancer* crabs throughout northern New England and the Canadian Maritimes (review in ^23^), reducing profits from the northwest Atlantic shellfish industry by as much as $22.6 million USD each year since its introduction ^38^. Studies from previous invasions of *C. maenas* suggest Puget Sound’s commercially harvested bivalve species are a likely target for green crab predation ^23, 38, 39^. Green crab invasion in Puget Sound is predicted to impact economically important hardshell clam, oyster and mussel species ^36^ all of which bring in millions of dollars in direct and indirect revenue to the Puget Sound region ^40, 41^.

In addition to the risk to shellfish, green crab risk assessments from Puget Sound and a field experiment in the northeast Atlantic have demonstrated the negative effects of green crabs on natural habitats; for example, *Zostera marina* eelgrass habitat is destroyed and associated food webs are disrupted by green crab invasion ^15, 42^. The loss of these habitats could have secondary impacts on many of the benefits that eelgrass provides to near-shore estuaries, such as essential spawning habitat for herring, and nursery grounds for many commercially important fish and shellfish ^43, 44^. Clearly, not all impacts of green crab are likely to be negative: Coho salmon ( *Oncorhynchus kisutch*) were predicted to benefit from invasion by feeding on the larvae of green crab, although this projection is associated with great uncertainty ^15^ and is not included in the current model.

Native to northern Europe, *C. maenas* has established populations in North America ^45^, South Africa ^46^, Japan ^47^, Argentina ^48^ and Australia ^49^. In North America, the green crab was first found on the northern Atlantic coast in 1899, and it has since expanded to cover 1000 kilometers of coast from Virginia in the south to a still-expanding range on Prince Edward Island ^45, 50^. In 1989, *C. maenas* is believed to have reached San Francisco Bay in the northeast Pacific via ballast water from populations on the northwest Atlantic coast ^51^.

**Figure 1.**
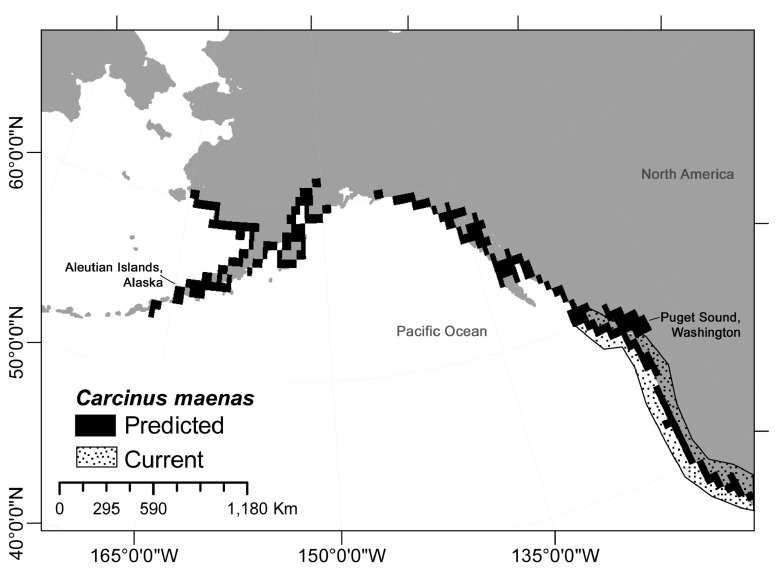
Potential *Carcinus maenas distribution* for the northeast Pacific coast from Northern California to Alaska, USA. The current nonnative distribution along the coast is indicated by a broad, stippled polygon, while the potential distribution of the species is plotted in black. Figure and MaxEnt potential distribution model from deRivera *et al.* ^83^, figure altered to clarify the absence of *C. maenas* from Puget Sound. This figure has been adapted and reproduced with kind permission from Diversity and Distributions, © 2011.

 Secondary spread of green crab north along the northeast Pacific coastline has been correlated with increased seawater temperatures and north-running coastal currents during the 1998 El Nino event, making it particularly likely that future climate change will allow the crab to invade new areas of coastline ^52, 53^. The green crab is limited by temperature and salinity, surviving in water temperatures ranging from 0°C to 30°C and salinities of 4 to 34 mV/V, although reproduction and larval survival occur in a more limited range than the adults (review in ^35^). Predictions based on these physiological limitations suggest that under current conditions green crab will continue expanding northward from its current northern extent of Vancouver Island until it reaches the Aleutian Islands ^45^, and that it may enter the contiguous waters of Puget Sound and the Strait of Georgia, BC, either by secondary introduction events from ballast release from large shipping freighters or through natural larval dispersal during one of the next El Nino events ( Figure 1) ^54^. Puget Sound is a large coastal estuary where extensive mudflats, eelgrass beds and warmer inland waters could provide optimal habitats for *C. maenas* foraging and reproduction.

### Biomass and revenue of Puget Sound’s shellfish harvest

**Figure 2.**
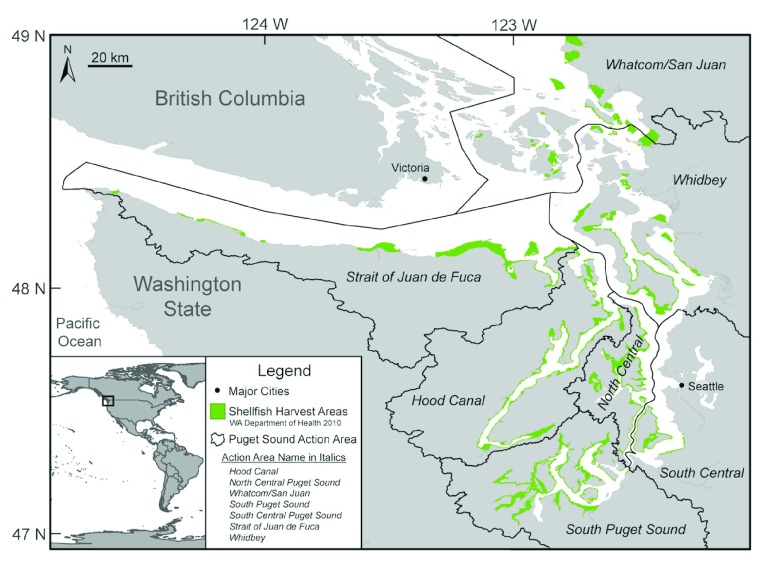
Puget Sound Action Areas and commercial shellfish growing areas. Boundaries of the Puget Sound Action Areas, each of which represent a unique watershed and harvest region as designated by the Puget Sound Partnership ^56^. Washington State Department of Health's approved commercial shellfish growing areas for 2010 ^57^ are highlighted in green. Included in this study are data on shellfish harvest biomass and revenue from all Action Areas except South Central Puget Sound.

To estimate the harvest for Puget Sound’s commercial shellfish industry, we obtained commercial harvest data for 2009 from PacFIN on May 26, 2010 ^55^. These data included species information for all clams, mussels and oysters commercially caught and farmed in Puget Sound, harvest biomass (kg) and total revenue (USD) for 2009 (summarized in Table 1). Data were apportioned to individual Puget Sound Partnership (PSP) action areas ^56^ ( Figure 2) by intersecting these with Washington Department of Fish and Wildlife (WDFW) and Department of Health 2010 approved commercial shellfish growing areas ^57^ using the ArcGIS 9 Intersect tool ( Figure 2; completed by Mark Plummer, NOAA). Data were assigned to PSP action areas assuming the harvest occurs uniformly throughout the growing area. Commercial data included harvest (kilograms year ^-1^) and total revenue (USD year ^-1^) for hardshell clams ( *Venerupis philipinarum* and *Protothaca staminea*), oysters ( *Crassostrea virginica* and *C. gigas*) and mussels ( *Mytilus* spp.) in six PSP action areas (hereafter, harvest areas). Shellfish biomass within each species group was summed to create a total estimated biomass for each harvest area. This biomass was then used to calculate an average cost per kilogram of shellfish (USD kg ^-1^). These data were used as the baseline estimate for current shellfish harvest and revenue before green crab invasion.

**Table 1.** Harvest of shellfish species a) hardshell clams, b) oysters, and c) mussels, by PSP action area in Puget Sound, size of harvested area (km ^2^), kilograms of shellfish harvested each year (in millions of kilograms year ^-1^; 10 ^6^ kg), average price per kilogram (USD/kg) (data from PacFIN) ^55^, and total revenue (in millions of dollars, USD M). Total kilograms of shellfish harvested and revenue in Puget Sound are summed as the grand total for all species, and listed with the average price for all three shellfish across the total harvest shellfish harvest.

Shellfish species	Action area	Area (km ^2^)	10 ^6^ kg	Avg. $/kg	Revenue (USD M)
a. Hardshell Clams	Hood Canal	126	0.880	$5.28	$4.65
	North Central Puget Sound	41	0.045	$3.97	$0.18
	Whatcom/San Juan	54	0.191	$4.46	$0.85
	South Puget Sound	108	2.118	$5.58	$11.82
	Strait of Juan de Fuca	104	0.041	$3.18	$0.13
	Whidbey	90	0.136	$5.22	$0.71
	*Puget Sound total*	*524*	*3.411*	*$5.38*	*$18.34*
b. Oysters	Hood Canal	126	0.567	$8.34	$4.73
	North Central Puget Sound	41	0.005	$2.20	$0.01
	Whatcom/San Juan	54	0.036	$11.85	$0.43
	South Puget Sound	108	0.694	$12.03	$8.35
	Strait of Juan de Fuca	104	0.014	$8.08	$0.11
	Whidbey	90	0.036	$11.85	$0.43
	*Puget Sound total*	*524*	*1.347*	*$10.44*	*$14.06*
c. Mussels	Hood Canal	126	0.001	$4.31	<$0.01
	North Central Puget Sound	41	0.000	$0.00	$0.00
	Whatcom/San Juan	54	0.002	$5.51	$0.01
	South Puget Sound	108	0.445	$4.61	$2.05
	Strait of Juan de Fuca	104	0.001	$7.35	$0.01
	Whidbey	90	0.386	$7.21	$2.78
	*Puget Sound total*	*483*	*1.288*	*$3.76*	*$4.85*
	**Grand total and average price**		6.046	$6.16	$37.26

The seven harvest areas of Puget Sound with complete commercial harvest data for each taxa group were Hood Canal, North Central Puget Sound, Whatcom/San Juan, Strait of Juan de Fuca, Whidbey Island, South Central Puget Sound, and South Puget Sound. South Central Puget Sound only represents 0.4% of the total harvest area and was not included in the analyses.

Shellfish species in Puget Sound are commercially harvested from mudflats or grown in aquaculture farms. All species evaluated in this study spend a portion of their life-cycle in the near-shore where they are susceptible to green crab predation:

a) Hardshell clams—Manila ( *V. philipinarum*) and native Littleneck ( *P. staminea*)—are harvested on tidal flats throughout Puget Sound at sediment depths of less than 15 cm. Hardshell clams are either raked off beaches where they grow naturally or their beds are “seeded” (seed clams are sown onto beaches leased from Washington State). These two species make up 98% of total hardshell clam harvest.
b) Oysters—European ( *C. virginica*) and Pacific oyster ( *C. gigas*)—are harvested from populations that grow without assistance in the high subtidal/low intertidal and on aquaculture farms where they are grown directly on mudflats, on racks sitting on the bottom substrate, or suspended under floating rafts.
c) Blue mussels ( *Mytilus* spp.) grow on rocks in the high subtidal/low intertidal and are harvested on state approved beaches and on aquaculture farms grown on racks sitting on the bottom substrate or suspended under floating rafts.

### Model of *Carcinus maenas* impacts on shellfish harvested in Puget Sound

To model the impact of *C. maenas* predation on shellfish in Puget Sound we applied the following simple model to estimate the total kilograms of each of three species of shellfish species groups (hardshell clams, oysters, mussels) consumed each year by *C. maenas* ( *Consumption*):

*Consumption* = ( *Area* × *Den* × *Cal* × *Diet*)/ *Cal kg ^-1^*

The variables *Area*, *Den*, *Cal*, *Diet*, and *Cal kg ^-1^* are defined below.

***Area***: Commercial shellfish harvest area (km ^2^) in Puget Sound ( *Area*) was estimated as 523.71 km ^2^ by tracing polygons around the shellfish harvest areas in the Washington State Department of Health Annual Inventory Growing Areas Map ^58^ within each of the five PSP action areas ( Figure 2) using ImageJ ( Table 1) ^59^.

***Den***: Density of adult *C. maenas* invading the harvest areas (crabs km ^-2^) was represented as a range of possible invasion densities. The high density estimate, 100,000 km ^-2^ (High), represents maximum adult green crab densities from one study in *C. maenas*’s native range, in Sweden ^60^. The high estimate for population density averaged over time is justifiable given that densities are sometimes considerably higher in invaded ranges due to lower predation pressures and greater available resources ^61, 62^. The low density estimate, one order of magnitude less than high at 10,000 km ^-2^ (Low), is equivalent to average adult green crab densities in their native range ^60^. We selected a medium density estimate of 50,000 km ^-2^ (Medium), roughly midway between high and low estimates. We refer to studies of densities from the native range because studies from invaded regions use catch per unit effort (CPUE) to estimate invasion densities ^33, 48, 63, 64^. CPUE is difficult to translate to a density of individuals per area because studies use different traps or dredges, set them using different methods, and leave them to catch crab for differing lengths of time.

***Cal***: The number of calories consumed by each adult *C. maenas* per year was estimated by extrapolation from laboratory diet studies. The number of calories mg ^-1^ (ash-free dry weight, AFDW) of shellfish meat from an individual adult shellfish (clams, 6.15 cal mg ^-1^; oysters, 4.85 cal mg ^-1^; mussels 5.47 cal mg ^-1^; as reviewed in ^65^) was multiplied by the number of mg per day a *C. maenas* (25 to 32 cm carapace) was found to consume. However, green crab prefer to prey on juvenile shellfish ^66, 67^ while shellfish harvest biomass is of adult shellfish, estimates based on harvested adult may overestimate predation impacts. In addition, this model assumes all harvested shellfish biomass is accessible to green crab predation. In practice, aquaculture using racks suspended above the bottom sediment to grow shellfish will limit or prevent predation, and clams may burrow deeper than green crab can dig through the sediment thus reducing predation on these species; on the other hand it is possible that deep-burrowing clams may be economically infeasible to harvest.

***Diet***: The proportion of the *C. maenas* diet consisting of each shellfish species group being modeled was estimated as ranging from 0.20 to 0.35. Grosholz and Ruiz ^37^ demonstrated that green crab diet is similarly dominated by bivalves in each region it has invaded. Our estimate range for diet assumes that 60–100% of *C. maenas’*s diet will be harvestable hardshell clams, oysters, and mussels, thus allowing for other species to comprise up to 40% of *C. maenas*’s diet ^37, 68^. Estimates of *Diet* do not include prey-switching by green crab, which may occur as the preferred shellfish biomass is reduced during invasion.

***Cal kg ^-1^***: We used calories mg ^-1^ AFDW (ash free dry weight) of shellfish meat (as in *Cal*; reviewed by ^65^), to calculate the number of calories per kilogram of shellfish ( *Cal kg ^-1^*). AFDW was converted to wet weight (WW), the unit of biomass for shellfish harvested in Puget Sound, using conversions reviewed in Ricciardi & Bourget ^69^. Conversion estimates were different for each species group and include a 95% confidence interval for all estimates that were made using more than one study. We used AFDW/WW conversion estimates of *M. edulis* for mussels (2.5–6.7), *C. virginica* for oysters (1.7), and the average conversion estimates of all bivalves for hardshell clams (5.2–6.4; combined for the two hardshell clam species).

We accounted for uncertainty in these parameters in two ways. First, where the range in potential data for the variable is large, as described below for crab density ( *Den*) and calorie diet for each crab ( *Cal*), we set three broad estimates (high, medium, and low) across the range of data from the literature and modeled these as separate scenarios for *C. maenas* consumption. Second, where the range in data uncertainty was smaller, we represented the uncertainty through randomization within scenarios defined as above, by choosing values across a uniform distribution for harvest area ( *Area*), the proportion of each crab’s diet that is the shellfish being analyzed ( *Diet*), and the number of calories per kilogram of each shellfish species ( *Cal kg ^-1^*). *Den* and *Cal* parameters are arguably not independent: certain values of one have the potential to constrain values of the other. This is an important possibility for which there is insufficient data to model the relationship given the presence of other complicating factors, such as limited recruitment of harvestable shellfish (see Discussion).

To implement the randomization for *Area*, *Diet*, and *Cal kg ^-1^*, we used a Markov Chain Monte Carlo (MCMC) algorithm using R software ^70^. Data were generated by resampling 10,000 times within the constraints described below for each parameter in the consumption model. To implement the continuous variation between the upper and lower bounds for *Area* and *Diet* parameters, we used the runif() function in R. Runif samples randomly from a uniform distribution between the upper and lower bound parameters. For *Area* we assumed this range to be relatively small around the single estimate above, ± 75 km ^2^, as some error was possible as a result of apportioning of WDFW Shellfish Management and Aquaculture areas to PSP action areas and the measurement of harvest areas using ImageJ. The *Diet* proportion was evenly sampled between 0.20 and 0.35 for each shellfish species *.* Considering *C. maenas* has not yet invaded Puget Sound nor had its predation preference tested for any of these shellfish species, precise estimates of its diet preference with the species it will encounter in the Puget Sound region were not available. In addition, these values will likely vary depending on the availability of shellfish and ease of predation. We attempted to partly reflect this by allowing diet preferences values to vary evenly across a range of proportions. We calculated the range in number of calories per kilogram of each shellfish species using the range of conversion estimates between AFDW and WW for each shellfish species group ( *Cal kg ^-1^*). An example of the R code used for these analyses is available in the Supplementary Materials.

We estimated the impact of green crab on harvested shellfish for high, medium, and low invasion densities at high, medium, and low calorie diets by calculating the error for mean consumption at each density (95% confidence interval from randomizations within each scenario). This calculation resulted in an upper and lower estimate of consumption of total shellfish biomass in harvest areas for each combination of invasion density and calorie intake. Annual harvest of each shellfish species was then estimated as the total baseline annual harvest minus the upper and lower consumption estimates. These methods were repeated for each of the three shellfish species groups: hardshell clams, oysters and mussels.

In order to represent parameter combinations that fall between the consumption scenarios for *Den* and *Cal*, we performed a partial sensitivity analysis of parameters in the model ^71^. In this analysis, we allowed *Den* and *Cal* to range freely from zero to high estimates, with a uniform distribution, and held the other parameters to the same constraints as described above, except *Diet* of harvested shellfish was estimated as 60% to 100% of green crab diet to include all three harvested shellfish species groups. Data were again sampled with the MCMC algorithm using R software ^70^, with 10,000 replicate samples within the parameter constraints.

### Estimating impacts on shellfish value in Puget Sound

We considered the primary economic value of shellfish harvest in terms of existing harvest revenue (landed value) and secondary economic value in terms of processing and distribution value and direct impacts from primary and secondary value in terms of labour income and employment. To evaluate the primary economic value-at-risk from green crab predation on shellfisheries in Puget Sound, we estimated the loss of existing harvested revenue from the total revenue of hardshell clams, oysters and mussels for high, medium, and low densities of green crab and across high, medium, and low calorie diets. Loss of shellfish harvest revenue, which is calculated as USD kg ^-1^, was assumed to decrease in parallel with the loss of harvested shellfish biomass to green crab predation as estimated by the consumption model. While estimates of net profit would also be a useful indicator of value-loss as they include expenses, many costs are expenditures that fuel the local economy (especially workers wages) and including them would be of interest to policymakers and politicians, thus, estimates of revenue are appropriate for estimating changes to the value of shellfish harvest.

Further estimates of the secondary economic value from direct impacts of Puget Sound shellfisheries: processing, distribution, and labour values, were made using benefit transfer methods as data on secondary value were not available for shellfish species in Puget Sound. Thus, we compared the known revenue of Puget Sound’s shellfish harvest to an analysis done on the value of shellfish harvest in BC, an adjacent region to Puget Sound ( Table 2) ^72^. GSGislason & Associates Ltd. ^72^ estimated the value of all of BC’s shellfisheries (in CAD) in 2005 for harvesting of shellfish, which involves the use of beach harvest, diving and other gear and aquaculture of shellfish from seed to market size. Secondary economic values were estimated for all fisheries species (including both fish and shellfish). To calculate direct impacts of shellfisheries alone, we assumed the ratio of the secondary value as compared to harvesting value of shellfisheries was the same as the ratio of the secondary value as compared to the fish and shellfisheries harvesting value.

**Table 2.** Projected economic values for shellfish harvest and processing, under three scenarios each for green crab densities and caloric intakes. The % shellfish harvest value revenue losses (in parentheses) were used to calculate the loss of shellfish value in processing estimated from green crab invasion, while distribution value was calculated as 15% of harvesting and processing margin. Values calculated for Puget Sound were made using benefit transfer from British Columbia in 2005 ^72^.

			Crab densities		
a. Low calorie diet	BC	Puget Sound	Low	Medium	High
*i. Primary & Secondary Value*			(0.2%)	(0.6%)	(2.76%)
Harvesting ^b^ (USD M)	139	37.3 ^a^	37.2	37.0	36.2
Processing Margin (USD M)	71 ^c^	19.1 ^d^	19.0	18.9	18.5
Distribution Margin (USD M)	32 ^e^	8.4 ^e^	8.4	8.4	8.2
*ii. Direct Impacts*					
Labour Income (USD M)	95 ^f^	25.5 ^g^	25.4	25.3	24.8
Employment (PYs)	2580 ^f^	692 ^g^	690	687	673
b. Medium calorie diet	BC	Puget Sound	Low	Medium	High
*i. Primary & Secondary Value*			(3.7%)	(10.0%)	(37.1%)
Harvesting ^b^ (USD M)	139	37.3 ^a^	35.9	33.5	23.4
Processing Margin (USD M)	71 ^c^	19.1 ^d^	18.3	17.1	12.0
Distribution Margin (USD M)	32 ^e^	8.4 ^e^	8.1	7.6	5.3
*ii. Direct Impacts*					
Labour Income (USD M)	95 ^f^	25.5 ^g^	24.5	22.9	16.0
Employment (PYs)	2580 ^f^	692 ^g^	666	623	435
c. High calorie diet	BC	Puget Sound	Low	Medium	High
*i. Primary & Secondary Value*			(6.8%)	(18.1%)	(63.9%)
Harvesting ^b^ (USD M)	139	37.3 ^a^	34.7	30.5	13.5
Processing Margin (USD M)	71 ^c^	19.1 ^d^	17.8	15.6	6.9
Distribution Margin (USD M)	32 ^e^	8.4 ^e^	7.9	6.9	3.1
*ii. Direct Impacts*					
Labour Income (USD M)	95 ^f^	25.5 ^g^	23.7	20.8	9.2
Employment (PYs)	2580 ^f^	692 ^g^	645	566	250

a. Puget Sound harvest, as calculated for *Total* Revenue in Table 1

b. Harvesting value at low, medium and high crab densities for each calorie diet as calculated in Table 2

c. Processing margin in BC, calculated as wholesale minus landed/farm value ( ^72^ page 12) for capture and aquaculture shellfish

d. Processing margin in Puget Sound, calculated as the same ratio as BC’s harvesting/processing margin (51%)

e. Distribution margin for BC and Puget Sound was estimated as 15% of harvesting plus processing margin ( ^72^ page 14)

f. Labour income and employment for BC shellfish calculated as 20% of BC total fisheries, the difference between total fisheries landed/farm value and shellfish only landed/farm value ( ^72^ page 12), values taken from direct impacts ( ^72^ page 14)

g. Labour income and employment for Puget Sound shellfish calculated as 27% of total fisheries employment, the difference between shellfish harvested value in Puget Sound/BC

Secondary values were calculated for 1) the processing margin, which includes transportation from sea to processing plants and the processing of raw shellfish, 2) the distribution margin, defined as the delivery of these processed shellfish products to consumers through wholesale and retail food channels, 3) the direct impacts of the seafood industry on labour income, which includes annual wages, salaries, and employer contributions to health and dental plans, pension plans, etc., and 4) employment years in persons per year (PY) ^73^. To estimate the secondary value of shellfisheries in Puget Sound, we then assumed the ratio of secondary economic value to shellfish harvest revenue is the same for shellfisheries in Puget Sound as in BC ( Table 2). This ratio was also assumed to be the same for commercial shellfish processing, distribution, labour income and employment in Puget Sound. We then estimated *C. maenas* impact on these secondary economic values at high, medium, and low invasion densities and high, medium, and low calorie diets. We assumed the loss of shellfish harvest revenue and secondary values decreased at the same rate as more green crab invade and consume increasing calories per crab as estimated by the consumption model.

## Results

### Commercial shellfish harvest

The baseline for total shellfish harvest in Puget Sound for 2009 for hardshell clams, oysters and mussels recorded by PacFIN ^55^ was 6.05 million kg of shellfish, with a landed harvest value of $37.26 million USD ( Table 1). Hardshell clams had the highest biomass harvested out of the three species groups (3.4 million kg) and the greatest associated revenue, $18.3 million USD, even though oyster species are valued per kilogram at almost twice that of hardshell clams, $10.44 kg ^-1^ compared to $5.38 kg ^-1^ respectively (price does not include the shell).

### Commercial shellfish harvest at risk from green crab invasion

**Figure 3.**
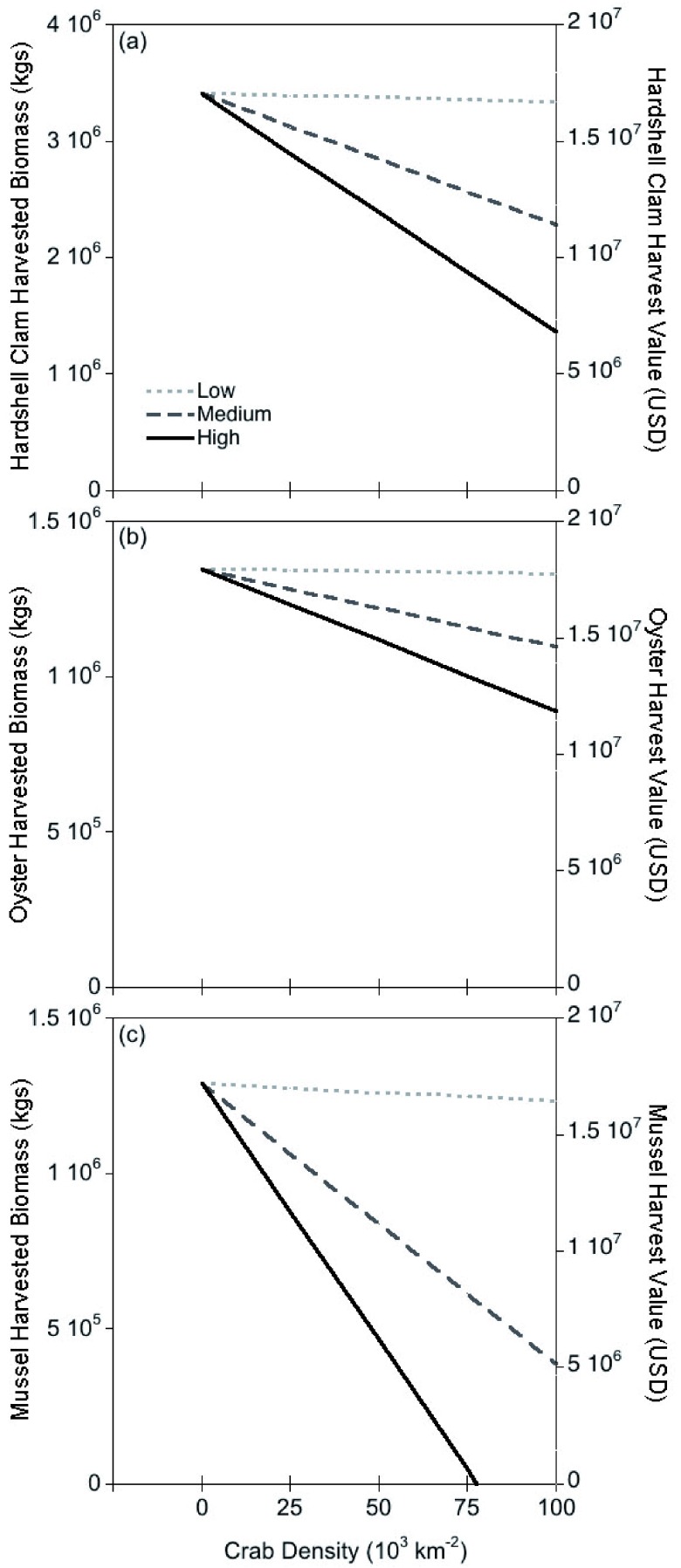
Loss of shellfish biomass and revenue at increasing densities of green crab. Mean harvested shellfish biomass (kg) and revenue (USD) at increasing levels of green crab densities for three shellfish species groups: **a**) hardshell clams, **b**) oysters, **c**) mussels at three increasing levels (dotted light grey, low; dark grey dashes, medium; black solid line, high) of calories consumed by each green crab year ^-1^.

Using the consumption model, we estimated harvested shellfish biomass and total shellfish harvest revenue associated with scenarios of low, medium and high calorie diets and densities for green crab: the medium-medium ( *Cal*- *Den*) scenario yielded a value-at-risk estimate of 0.54 million kg and $3.72 million USD in harvest revenue, a loss of 9.0%; the low-low scenario suggested a minor loss of only 0.04 million kg and $0.08 million USD, a loss of 0.3%; and medium-high, high-medium, and high-high scenarios suggesting losses of at least 0.99 million kg and $6.76 million USD ( Table 3 and Figure 3). Mussels were the only shellfish to reach an estimated value loss of 100% for the high-high scenario ( Figure 3c). Note that these losses pertain to losses of harvested biomass, not of all prey organisms, allowing for the possibility that green crab might eliminate harvestable biomass without causing local extirpation (see Discussion).

**Table 3.** Shellfish harvest before and after green crab predation in Puget Sound. The baseline shellfish biomass harvest (millions) and direct harvest revenue value (millions, 2009 USD) are compared to shellfish harvest under three scenarios of green crab densities (low, medium, and high densities) and of calories consumed by green crab each year (low, medium, and high calorie diets).

			Crab densities					
a. Low calorie diet	Baseline		Low		Medium		High	
Species	10 ^6^ kg	$M	10 ^6^ kg	$M	10 ^6^ kg	$M	10 ^6^ kg	$M
Hardshell Clams	3.41	$18.30	3.40	$18.31	3.40	$18.29	3.34	$17.97
Oysters	1.35	$14.10	1.33	$14.04	1.33	$13.93	1.32	$13.80
Mussels	1.29	$4.80	1.28	$4.83	1.28	$4.81	1.23	$4.64
*Total*	*6.05*	*$37.26*	*6.01*	*$37.18*	*6.01*	*$37.03*	*5.90*	*$36.23*
	**Change from baseline**		**0.04**	**$0.08**	**0.04**	**$0.23**	**0.15**	**$1.03**
b. Medium calorie diet	Baseline		Low		Medium		High	
Species	10 ^6^ kg	$M	10 ^6^ kg	$M	10 ^6^ kg	$M	10 ^6^ kg	$M
Hardshell Clams	3.41	$18.30	3.30	$17.74	3.24	$17.44	2.28	$12.28
Oysters	1.35	$14.10	1.31	$13.62	1.14	$11.88	0.93	$9.69
Mussels	1.29	$4.80	1.20	$4.51	1.12	$4.22	0.39	$1.46
*Total*	*6.05*	*$37.26*	*5.80*	*$35.87*	*5.50*	*$33.54*	*3.60*	*$23.43*
	**Change from baseline**		**0.24**	**1.39**	**0.54**	**$3.72**	**2.45**	**$13.83**
c. High calorie diet	Baseline		Low		Medium		High	
Species	10 ^6^ kg	$M	10 ^6^ kg	$M	10 ^6^ kg	$M	10 ^6^ kg	$M
Hardshell Clams	3.41	$18.30	3.20	$17.24	3.11	$16.70	1.37	$7.35
Oysters	1.35	$14.10	1.27	$13.27	0.97	$10.10	0.59	$6.11
Mussels	1.29	$4.80	1.12	1.12	0.98	$3.70	0.00	$0.00
*Total*	*6.05*	*$37.26*	*5.60*	*$34.74*	*5.06*	*$30.50*	*1.95*	*$13.46*
	**Change from baseline**		**0.45**	**$2.52**	**0.99**	**$6.76**	**4.46**	**$23.80**

**Figure 4.**
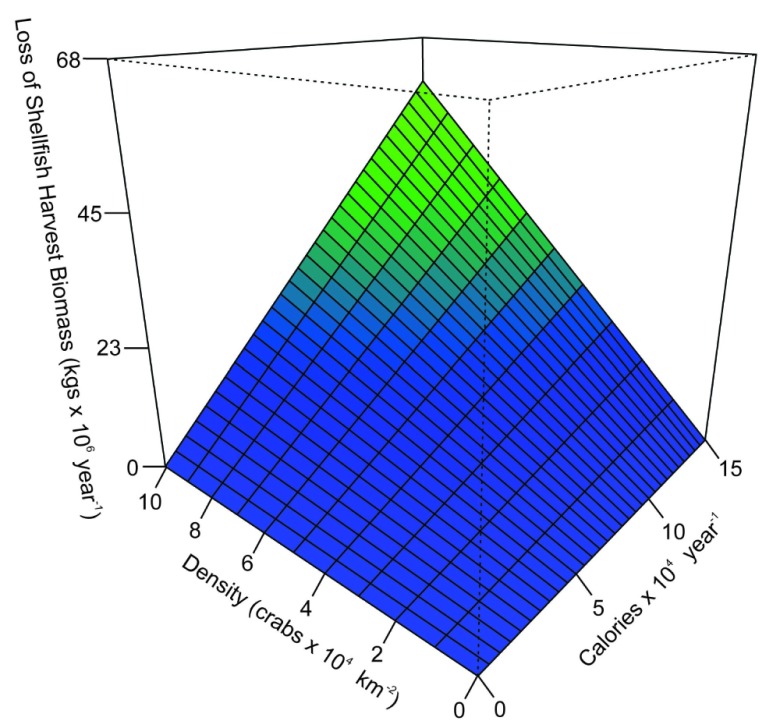
Sensitivity analysis of the loss of shellfish harvest biomass. Loss of shellfish harvest biomass (kg year ^-1^) as a function of calories consumed each year and the density of *C. maenas* km ^-2^ in harvest areas. Assumes 60% to 100% of green crab diet is made up of hardshell clams, oysters and mussels.

The Monte Carlo sensitivity analysis demonstrated the relationship between density and calorie consumption rate on projected loss of shellfish harvest biomass ( Figure 4). If crabs invade at high densities but consume calories at a low rate, or vice versa, they would likely have a limited effect on shellfish harvest. However, at high densities and high calorie intake rates the slope of the shellfish harvest loss is steep and green crab will likely greatly reduce shellfish harvest biomass.

### Value-at-risk associated with commercial shellfish harvest loss

The harvest revenue associated with the total biomass for these three species of shellfish in Puget Sound was $37.26 million USD (26.6% of BC’s revenue for 2009). Estimated loss at low, medium and high green crab calorie diets was up to 3%, 37% and 64% at the highest green crab density of 100,000 green crab per km ^2^. Specific values for change in known harvesting values and estimated processing margin and distribution margin values, as well as potential change in labour income, are presented in Table 2. Employment associated with shellfish harvest and farming, processing, and distribution is estimated around 692 person-years per year (PYs). Green crab invasion at highest densities is estimated to reduce these PYs by 47 at the lowest calorie diet, and by 442 at the high calorie diet ( Table 2).

## Discussion

 By assessing the value-at-risk for commercial shellfish harvest in nearshore ecosystems of Puget Sound, Washington from the green crab, *C. maenas* we estimated a range of possible losses each year that included, at highest invasion densities for three scenarios of calorie-intensity of diets, up to 0.15–4.46 million kg year ^-1^ of shellfish worth $1.03–$23.8 million USD, a loss of 2.8–64%. For highest calorie diets, across a range of invasion densities shellfish harvest loss estimates ranged from 0.45–4.46 million kg year ^-1^ worth $2.5–23.8 million USD (6.8–64% loss). In addition to loss of harvest, processing margins were estimated to be reduced by $1.3–12.2 million USD and distribution by $0.6–5.3 million USD, all resulting in a loss of 47–442 jobs (PYs). Estimated value-at-risk is likely to vary across Puget Sound harvest areas due to refuges for shellfish populations and uneven distributions of green crab due to habitat variability. Green crab appears to have the potential to reduce shellfish biomass enough to effectively halt the shellfish harvest industry completely at the highest invasion densities or highest calorie diet rate, since low shellfish population densities result in shellfish harvest area closures.

 If green crab significantly contributes to reducing biomass, hardshell clam populations may be reduced below allowable harvest biomass. Thus, reducing populations of harvested shellfish to zero within the consumption model does not suggest there are no shellfish remaining, but rather that when shellfish populations are sufficiently reduced there is no more commercial harvest of shellfish. In addition, green crab are opportunistic feeders that are likely to feed on other species when preferred bivalve abundance becomes low. So although green crab may decrease shellfish population densities, they are likely to feed on whatever species are the easiest to access, maintaining bivalve populations at low densities but not extirpating the populations ^64^. The reduction in shellfish biomass may also increase the growth and survival of juvenile shellfish by reducing competition for space and food; while at the same time, biomass loss has the potential to limit recruitment of shellfish by reducing the population of reproductive adult shellfish and by preying on newly settled juvenile shellfish. These negative feedbacks may result in greater losses to shellfish then we have estimated.

 Although the range in values is broad it appropriately represents the uncertainty in key factors associated with green crab invasion impacts, factors that are highly variable across regions. As such, the most extreme estimates of shellfish harvest loss cannot be wholly excluded from consideration: these estimates are based on current understanding of green crab populations in other regions, with several caveats. Lower calorie estimates are likely more similar to impacts from previous invasions than high calorie estimates because when green crab invade at high densities, they are unlikely to have unlimited access to shellfish. That is, high densities of green crab invasion will reduce harvested shellfish biomass through predation, however if there is intraspecific competition for prey then high crab densities are likely to reduce the number of calories consumed per crab ^74^. Thus, it is unlikely that the highest crab invasion densities will consume calories at the highest rates. The lower calorie estimates demonstrate a limited access scenario and therefore present a more realistic change in biomass. Yet, significant estimates of possible shellfish harvest loss are reached both at high calorie diets but medium crab densities, and at high crab densities but medium calorie diets, suggesting that there are multiple plausible invasion scenarios that could result in a loss to shellfish harvest.

 The lack of previous data on economic loss to green crab makes it difficult to compare our estimates of value-at-risk in Puget Sound to losses experienced elsewhere. In the northeast Atlantic, the shellfish industry was estimated to have lost 4.5 million kg of biomass and $22.6 million USD in revenue each year from green crab, though this estimate did not include oyster species, potentially underestimating total costs, and did not describe what proportion of the total fishery was lost to green crab ^38^. Without knowing what the total biomass of this northeast Atlantic fishery is, it is not possible to compare the estimates of value-at-risk to green crab invasion as calculated in this study. This same study estimated the cost of green crab’s future predation impact in the northeast Pacific at $844,000 USD year ^-1^ once green crab expand into Puget Sound and up to the Aleutian Islands ^38^. This coarse estimate may be low as it does not include regional variation in feeding rate or all the harvest shellfish species at risk, though it does estimate costs across increasing invasion densities. However, total Washington State shellfish harvest value was underestimated as $17.2 million USD year ^-1^ for the entire state (not including oysters) ^38^, while in our study we demonstrated that harvest of hardshell clams and mussels in Puget Sound alone was worth $23.2 million USD year ^-1^. Additional value-at-risk estimates for regions already invaded by green crab would improve estimates for not yet invaded regions.

 Not included in our estimates is a prediction of how change in shellfish biomass supply will affect willingness to pay for those shellfish (‘price elasticity’). If there is a strong ‘local’ aspect to the demand (a greater willingness to pay for Puget Sound shellfish), reductions in shellfish harvest of 60% would likely trigger an increase in market price. However, it is also likely that as costs increase, consumers will choose to eat shellfish produced in other regions that continue to produce low costing shellfish.

Recreational shellfisheries in Puget Sound are also likely to be directly affected by loss of shellfish biomass. As number of recreational harvest days per year decreases, harvesters are likely to experience reduced shellfish harvest and loss of cultural benefits, such as family engagement and traditional harvesting by native coastal tribes. Indirect effects may result when local human communities located near harvest beaches experience reduced benefits as fewer harvesters buy supplies, permits, food and lodging. The social and environmental-engagement value of recreational shellfish harvest might be difficult to quantify but is an important aspect of harvest value that is often ignored ^75^.

### Additional ecosystem changes

 The introduction of *C. maenas* is a threat to biodiversity and ecosystem function for Puget Sound’s near-shore food web ^23, 38, 39, 76^. The diet preference for bivalves and resulting ecological impact has been relatively similar across invaded regions ^37^. Assuming this remains true for Puget Sound, removal of bivalves by green crab may have indirect effects on shorebird populations by removing their prey, as seen in other sites in the northeast Pacific ^37^. Similarly, bivalve removal may also indirectly affect native crab populations resulting in prey switching and reduced biomass ^36^. Loss of native crab may reduce predation pressure on harvestable shellfish, potentially effecting estimates of shellfish biomass loss. Green crab bivalve predation also may result in a shift in the bivalve community if bivalve species that are less-preferred by green crab replace those consumed more heavily ^77^. When this occurred in Bodega Harbor, California, green crab suppressed the native clam, *Nutricola* spp., which had an indirect positive effect on the nonnative clam, *Gemma gemma*. Green crab also have the potential to increase tertiary productivity in Puget Sound, because their larvae provide a prey resource to fish species ^15^ and adults are likely to become prey for birds, seals, and fish that normally feed on local crab species.

 Shellfish threatened by this invasion provide more than just commercial and recreational harvest revenue. By filtering toxins and nutrients from Puget Sound, shellfish help to increase oxygen levels and reduce toxin accumulation in other organisms. Eutrophication in Hood Canal and southern Puget Sound is likely to increase with a decline in shellfish populations ^78^. Because these nonmonetary values are not incorporated in model estimates, this study likely underestimates the total value lost to green crab invasion.

### Motivating prevention and mitigation of invasive impacts

Managers can prepare for major losses of harvest and revenue by initiating strong preventative measures ^3, 13, 25^. Current efforts in Puget Sound to prevent green crab invasion include restricting out-of-state imports of shellfish, encouraging commercial shellfish harvesters to inspect their equipment before transferring gear between invaded and non-invaded regions, requiring ballast exchange before entering Puget Sound, and instituting a detection program that incorporates community volunteer groups and paid specialists ^79^. These efforts are useful but could be improved if more funding was allocated to preventative efforts. For example, funds could be used to better enforce gear inspection and ballast exchange. At present, ballast exchange is not required between Oregon, Washington, and BC, though green crab are already present along the outer coast of each of these states/provinces ( Washington Department of Fish and Wldlife (WDFW) ballast water program) ^80, 81^. In addition, increasing the number of paid specialists sampling for green crab would increase the chances of catching green crab invasion early and prevent further spread in Puget Sound ^82^.

Despite current efforts to limit human introduction of green crab into Puget Sound, climate change resulting in warming sea waters and changing current flow will likely result in larval transport into Puget Sound without further human assistance ^33, 83^. Plans to mitigate the impact of green crab will be most effective if they are in place before invasion occurs. Managers can prepare commercial harvesters to take preemptive measures in reducing the green crab’s impact by altering their current methods of shellfish aquaculture. Oyster and mussel racks suspended above the bottom substrate may limit green crab’s access to commercially cultured bivalves and this is already a common practice in neighboring BC (BC Shellfish Grower's Association Shellfish industry encyclopedia) ^84^. Anti-predator netting has been used successfully in the northeast Atlantic, reducing loss of clam biomass to predation by 13–55% ^85^. Netting may be used to minimized predation on Puget Sound clams and has been tested in BC, though this method has detrimental side-effects on other infaunal species also netted in the mudflat ^86^. A comprehensive post-invasion plan would combine measures to mitigate impacts with monitoring and control efforts ^25, 87^.

### Incorporating uncertainty of future invasion impacts

 Future research using the consumption model presented in this study can improve upon these estimates of green crab impact by incorporating greater detail on predation rates, shellfish recruitment effects, the variation of predation rate and calorie intake over time, spatial differences in invasion effects, and estimates of total biomass of shellfish in Puget Sound. The impact on each shellfish species could take into account both green crab preference for these prey species, their probability of encountering each species in the field, and density effects on predation rates. Future estimates may also benefit from considering the decrease in predation rate as total shellfish biomass in all of Puget Sound declines due to biomass loss and reduced recruitment of juvenile shellfish. In general, estimates of green crab invasion densities would be improved if future studies that sample existing populations of green crab included crab densities measured in a metric that is repeatable across regions, such as crab density per square meter measured using dredges or quadrats. While CPUE is useful for comparing densities within and between regions in a single study, measurements are difficult to translate into a density of crab per area and results may vary with trap type and deployment methods used.

It is not possible to precisely estimate the invasion impacts of green crab and other invading species because of temporal and spatial variation across invaded regions ^26– 29^ and the effect of management responses aimed at mitigating those impacts. However, though few data are available about specific impacts before invasion, researchers can refine estimates of value-at-risk by incorporating a range of potential invasion parameters: density of individuals, number of arrivals, potential predation and competition interactions, and economic impacts, these general estimates can produce more accurate assessments of what is known regarding the invading species ^12, 25^. If estimates are too simplistic and do not include uncertainty measures or are not made at all there is little motivation to rationalize economic spending to monitor, prevent or mitigate for species invasions before invasion occurs ^19^.

## Conclusions

At high densities or high calorie predation rates green crab may have the potential to reduce revenue from shellfish harvest and processing by as much as $1.6–41 million USD each year, representing real uncertainty in possible impacts. Value lost to shellfish harvest is dependent on the density of crabs that invade harvest areas and the actual calories consumed by each crab, parameters that are likely to be a product of the individual rate of predation and the accessibility to prey. Loss of shellfish has implications for recreational shellfish harvest and the potential for reduced filtration rates that may lead to increased eutrophication in already threatened coastal habitats ^88^. Preventing or reducing the effect of high-impact species invasions should be considered a priority, and the cost of action weighed against other management alternatives, as these invasions have direct economic impacts and a range of indirect effects on ecosystem function ^89, 90^. By incorporating uncertainty when estimating impacts from invasions, management plans can describe the range of potential costs of invasion and motivate preventative action in order to prepare for future ecosystem damage even when local impacts are still unknown ^12, 13, 25, 87^.

## Supplementary materials

**Green crab consumption model R code**. Click here to access the code. doi: 10.5256/f1000research.1014.s36713 ^91^.
