# Supplementary material for: Trading green backs for green crabs: evaluating the commercial shellfish harvest at risk from European green crab invasion
Source: F1000Res. 2014 Oct 16;2:66. Originally published 2013 Feb 28. [Version 3] doi: 10.12688/f1000research.2-66.v3 (PMC4231929; doi:10.12688/f1000research.2-66.v3)
Supplement: Peer review history

# Peer review: Referee response for version 3

**DOI:** 10.5256/f1000research.5883.r6628

**Published:** 2014-11-04

**Authors:** Elizabeth Petersen

**Affiliations:** 1 School of Agricultural and Resources Economics, University of Western Australia, Crawley, Australia

The authors have done a thorough job at addressing my comments and I am happy to recommend that the paper be approved for indexing.

I have read this submission. I believe that I have an appropriate level of expertise to confirm that it is of an acceptable scientific standard.

# Peer review: Referee response for version 2

**DOI:** 10.5256/f1000research.3989.r5667

**Published:** 2014-09-10

**Authors:** Elizabeth Petersen

**Affiliations:** 1 School of Agricultural and Resources Economics, University of Western Australia, Crawley, Australia

**Title: **

- When I first read the title, I found it confusing. It wasn't until I read the abstract that I understood the purpose of the paper. I suggest changing the word 'to' to 'from', 'due to' or 'of'.

**Abstract:**

- Clear and concise, a good summary of the article

**Introduction: **

- Well written and covers the relevant introductory subject matter.
- I suggest a couple of changes to Paragraph 5: Firstly, the second last sentence does not make sense. Maybe change it to something like "For example, if early preventative efforts had been funded to prevent invasion of the rusty crayfish ..., $6 million USD in fisheries harvest revenue during the 30 years since invasion may have been protected... I also suggest changing the word 'aligning' in the last sentence to something like 'prioritising'.
- I suggest one change to Paragraph 6: Merging the first two sentences.

**Methodology, results and discussion:**

- These sections are well written and deal well with uncertainties associated with predicting invasion impacts *a piori*. I have one concern. I don’t understand how the authors have dealt with invasion consequences over time. Usually, invading populations follow a logistic function over time. From what I read, the authors have calculated the cost at full invasion, rather than the costs over time of growing green crab populations. I am not sure whether the values for reduced revenues are annual values, or total values over a time period. If they are annual values, and if I have not misunderstood the study, the authors need to present them as year ^-1^ values and justify why they did not consider a net present value over time. If they are values that consider invasion over time, the authors need to clearly specify the time period and their discounting methodology.
- Table 1: I suggest removing the grand total value “$6.16” from the Avg. $/kg column and including it a new row named something like “Weighted average”.

**Conclusions:**

- I suggest the conclusions specify clearly that there will be costs associated with preventing or containing invasion, and that these results need to be considered by government and industry in the knowledge of whether it is most cost-effective to fund prevention of green crab invasion or other more pressing alternatives. I am not sure the authors can state “Preventing or reducing the effect of high-impact species invasions should be a priority as these invasions have direct economic impacts and a range of indirect effects on ecosystem function”. Perhaps it can be altered to something like “Preventing or reducing the effects of high-impact species invasions should be considered by government as a potential priority, as these invasions … “

I have read this submission. I believe that I have an appropriate level of expertise to confirm that it is of an acceptable scientific standard, however I have significant reservations, as outlined above.

# Peer review: Referee response for version 2

**DOI:** 10.5256/f1000research.3989.r4271

**Published:** 2014-03-27

**Authors:** Ryan Chisholm

**Affiliations:** 1 National University of Singapore, Kent Ridge, Singapore

I have looked over the revised manuscript and the authors' responses to my earlier comments, and I am happy with the changes.

I have read this submission. I believe that I have an appropriate level of expertise to confirm that it is of an acceptable scientific standard.

# Peer review: Referee response for version 1

**DOI:** 10.5256/f1000research.1164.r2041

**Published:** 2013-10-09

**Authors:** Nicholas Bax

**Affiliations:** 1 Commonwealth Scientific and Industrial Research Organisation, Tasmania, Australia

I personally have some level of discomfort with looking at the risks of invasions (or other environmental problems) solely from the point of view of consequences (called economic value at risk here) without also considering likelihood. This has been addressed to some degree in the introduction, but I think needs to be further emphasized both in the introduction and the discussion/conclusions.

One reason for including likelihood is that it reduces the danger that a result can be considered alarmist and dismissed. The well-known human failings in assessing risk by focusing on consequences rather than likelihood is very well discussed in the literature. Examples include the risk of a nuclear accident being perceived as of greater risk to an individual’s health than smoking. A second reason is that including probabilities moves the assessment from qualitative, or semi-quantitative (as in this case) to fully quantitative, and this tends to require a more systematic consideration of the problem.  

There are two major areas where I think the papers needs modifying, one of which, if I have interpreted the methods correctly, would reduce estimated losses by about 2/3:

 

1. There is no discussion of earlier estimates of the impacts of the green crab on Puget Sound shellfish (and other) resources – e.g. Lafferty and Kuris, 1996 (Ecology), or costs of marine invasives in general (e.g. Williams and Grosholz, 2008 (Estuaries and Coasts).)
2. The assumption that green crab would have a diet of 60-100% harvestable shellfish, is not well supported by any literature provided, but will directly affect the conclusions. The approach taken of simply adding the predation estimates for each species together is not realistic. One could equally argue that they are interchangeable and that shellfish (e.g. because of handling time?) would never form more of the diet than that represented by one species. At the individual crab level, each crab would have to move between beds or farms of the three species every day. If they did not, and stayed in one area as seems likely, they would only have the opportunity to eat one of the three shellfish species each day (week? month? year?). So I believe that adding them together is unrealistic.

I would also like to see the following points addressed:

1. It is assumed that a green crab invasion would be uniform, through time and space, in Puget Sound. How realistic is this?
2. There seems to be no allowance for the influence of predator-prey size ratios on the ability of green crabs to attack their prey. What effect will this have? Is there a size beyond which shellfish are protected?  Could rearing practices reduce the impact of predation?
3. What is the impact of reduced prey numbers on the growth and survival rate of the remaining shellfish?
4. What is the impact of the green crab on reducing existing predation from crabs that it will displace?
5.  There is no discussion of price elasticity in supply. Would prices of shellfish stay the same if supply dropped by 60% as suggested?
6. I did not note any discussion of expenses. Net profit would be a more informative indicator than revenue.
7. The numbers in Table 2 for direct loss of shellfish (0.2 to 55.9%) do not seem to agree with those in Table 3 (0.3 to 68.7%), or in those in the text describing the results.

I have read this submission. I believe that I have an appropriate level of expertise to confirm that it is of an acceptable scientific standard, however I have significant reservations, as outlined above.

# Response

**Published:** 2014-02-28

**Authors:** Megan Mach

**Affiliations:** Center for Ocean Solutions, Stanford University, USA

Dr. Bax,

Thank you for taking the time to review our article, we believe it is much improved by your comments. I have included our responses and notes on the changes made to the manuscript (in normal font) as a result of your comments (denoted in *italics*).

1. *"In response to comments regarding additional discussion of likelihood in the introduction and discussion:"*
  We agree that the issue of likelihood is critical, which is why we consider such wide ranges of values across several parameters. We include both high and low estimates of green crab consumption that account for a broad range in calorie intake levels and invasion densities to demonstrate the huge uncertainty inherent in how green crab will interact and impact this new ecosystem. The literature was not sufficient to inform explicit likelihood values for each of these scenarios, so we simply explore a wide range and report results across that range. Thus, issues of likelihood are implicit in the structure of our entire analysis, just without the specification of relatively likelihood. For example, under scenarios where few green crab invade, their impacts are likely to remain relatively low (depending on calorie consumption estimates).
  We also discuss some aspects of likelihood in the introduction as well as the discussion. For example, in the discussion we describe green crab as an opportunistic feeder likely to switch species when abundances become to low and that intraspecific competition is likely to reduce calories consumed by each crab (page 15).
2. *"There is no discussion of earlier estimates of the impacts of the green crab on Puget Sound shellfish (and other) resources – e.g.* *Lafferty and Kuris, 1996 (Ecology)* *, or costs of marine invasives in general (e.g.* *Williams and Grosholz, 2008 (Estuaries and Coasts)* *.)"*
  On page 16 we discuss earlier estimates of green crab’s future predation impact in Puget Sound and up to the Aleutian Islands; estimates are likely low because they do not include any regional variation in feeding rate or include many of the harvested shellfish species at risk ( Lovell *et al.* ,2007). This same report underestimated Washington’s shellfish harvest value by almost 5 million USD when compared to the data used in our analysis.
  We did not include estimates of green crabs’ impact on annual value of oysters by Lafferty and Kuris (1996; Table 1 of their article) because their estimates include Oregon, the outer coast of Washington and Puget Sound. We did not have a way to estimate the Puget Sound threatened annual value alone. As an example of the results in the two studies--the Lafferty and Kuris study estimates that green crab will reduce oyster value by $20 mil in 1990 USD (~$33 million in 2009 USD) for Oregon and Washington, while we estimate total revenue in 2009 for oysters in Puget Sound alone to be ~$14 million.
  I have removed the freshwater Zebra mussel example and replaced it with a jellyfish example from the review by Williams and Grosholz (2008) to provide a marine example of an economically costly invasive species (first paragraph of the introduction, page 3).
3. *"The assumption that green crab would have a diet of 60-100% harvestable shellfish, is not well supported by any literature provided, but will directly affect the conclusions. The approach taken of simply adding the predation estimates for each species together is not realistic."*
  This estimate allows for other species to comprise up to 40% of *C. maenas*’s diet and that crabs can feed on any of the three shellfish species we are modeling (mussels, clams, & oysters). These estimates are supported by a study ( Grosholz and Ruiz, 1996) that found these shellfish species groups as ranging from 20 to 35% of the total green crab diet. Our model assumes that green crab would have a diet that is made up of 60-100% of the harvestable shellfish (all three species together) over the course of a year based on this study, as described under *Diet* in the Methods section on page 9.
  " *One could equally argue that they are interchangeable and that shellfish (e.g. because of handling time?) would never form more of the diet than that represented by one species. At the individual crab level, each crab would have to move between beds or farms of the three species every day. If they did not, and stayed in one area as seems likely, they would only have the opportunity to eat one of the three shellfish species each day (week? month? year?). So I believe that adding them together is unrealistic."*
  This model estimates all calories consumed by the range of densities of green crab in harvestable areas over the course of a year, assuming a range of calorie requirements of the crabs. It does not assume that individual crabs move across beds every day, but rather we estimate the entire invading population (in harvest areas).
4. *"It is assumed that a green crab invasion would be uniform, through time and space, in Puget Sound. How realistic is this?"*
  Treatment of invasion as uniform across time and space is a limitation of this model. We have made a point to only discuss regions that have data on harvest densities, and only discuss impacts to harvest value (in commercial shellfish harvest areas) to limit the spatial scale to which our model applies. We describe the huge variability in past invasions that make it difficult to assess how green crab invasion will impact Puget Sound shellfish harvest (page 19), this is another reason we incorporated such a broad range of possible values, and we have also added additional text under “Incorporating uncertainty in future invasion impacts” on page 19.
  "Future research using the consumption model presented in this study can improve upon these estimates of green crab impact by incorporating greater detail on …the variation of predation rate and calorie intake over time, spatial differences in invasion effects…”
5. *"There seems to be no allowance for the influence of predator-prey size ratios on the ability of green crabs to attack their prey. What effect will this have? Is there a size beyond which shellfish are protected?  Could rearing practices reduce the impact of predation?"*
  On page 9, where we discuss the number of calories consumed by each adult green crab (Cal) we discuss green crabs preference for juvenile shellfish and that the harvest of shellfish is calculated for adult shellfish. These estimates may result in overestimating predation impacts.
  Rearing practices could indeed reduce the impact of green crabs on juveniles. This is discussed at the end of this same paragraph on page 9 along with a discussion of how plastic behavior, such as burrowing depth may also reduce predation. We also discuss rearing practices on page 18-19 in the “motivating prevention and mitigation of invasive impacts” section.
6. *"What is the impact of reduced prey numbers on the growth and survival rate of the remaining shellfish?"*
  On page 15 we discuss how “the reduction of shellfish biomass [] has the potential to limit recruitment of shellfish by reducing the population of reproductive adult shellfish and by preying on newly settled juvenile shellfish.”
  We have added “The reduction in shellfish biomass may also increase the growth and survival of juvenile shellfish by reducing competition for space and food” to this same section.
  In addition, in the section “additional ecosystem changes” on page 17 we discuss that the ecological impact of green crab has been relatively similar across regions. Assuming this remains true for Puget Sound, the impact of a reduction in prey number may result in a shift in the bivalve community if a bivalve species that is less preferred by green crab replaces those consumed more heavily.
7. *"What is the impact of the green crab on reducing existing predation from crabs that it will displace?"*
  The impact of green crab on native crab likely includes 1) reduced prey availability through competition; 2) reduced native crab biomass through predation (discussed briefly on page 5) and competition for shelter; and 3) reduced biomass through loss of nursery habitats (discussed on page 6).
  We added additional discussion of the potential indirect impact of green crab on native crabs to page 17 under “Additional ecosystem changes.” There will likely also be an indirect impact on shorebirds (also discussed here). Reduced native crab biomass could potentially affect our estimates of the impacts of green crab as native crabs will remove less shellfish biomass from the harvest areas. We have added a comment to this effect on page 17.
8. *"There is no discussion of price elasticity in supply. Would prices of shellfish stay the same if supply dropped by 60% as suggested?"*
  We have now included a discussion (page 16) of change in supply. This is an excellent point.
  “Not included in our estimates is a prediction of how change in shellfish biomass supply will affect willingness to pay for those shellfish (‘price elasticity’). If there is a strong ‘local’ aspect to the demand (a greater willingness to pay for Puget Sound shellfish), reductions in shellfish harvest of 60% would likely trigger an increase in market price. However, it is also likely that as costs increase, consumers will choose to eat shellfish produced in other regions that continue to produce low costing shellfish.”
9. *"I did not note any discussion of expenses* *. Net profit would be a more informative indicator than revenue."*
  Both metrics appear to be of interest to different parties. For example, policymakers and politicians may be more concerned with revenues, because many costs are expenditures that fuel the local economy (especially wages, which are typically a large share of costs in more-developed countries). Unfortunately, as we do not have access to the costs at each stage (primary or secondary economic value), we had no real basis to estimate profits. We added a discussion to this effect on page 12.
10. *"The numbers in Table 2 for direct loss of shellfish (0.2 to 55.9%) do not seem to agree with those in Table 3 (0.3 to 68.7%), or in those in the text describing the results."*
  The percent loss in Table 3 was specific to the total shellfish biomass under various scenarios as compared to the baseline biomass. The percent loss in Table 2 is the total shellfish revenue value under various scenarios as compared to the baseline revenue. To make this less confusing I have removed the percent loss values from Table 3 and altered the text in the table legend of Table 2.

# Peer review: Referee response for version 1

**DOI:** 10.5256/f1000research.1164.r848

**Published:** 2013-03-18

**Authors:** Ryan Chisholm

**Affiliations:** 1 National University of Singapore, Kent Ridge, Singapore

This manuscript looks at the potential economic impacts of invasive European green crabs on the shellfish industry in Puget Sound. They come up with a range of dollar values for potential economic impacts, based on both economic and ecological underlying uncertainties.

The manuscript is mostly sound, although I have one concern relating to their treatment of “primary” and “secondary” values:

•    How are “Processing margin” and “Distribution margin”, which make up the secondary value in Table 2, calculated? I understand that you are just applying ratios from ref. 72, but I want to understand where these ratios come from (I looked at ref. 72 briefly, but it wasn’t easy to figure out). Does this secondary value represent the difference between the market value and harvested value of the fish?  In any case, you must be careful to avoid double-counting when you sum the primary and secondary values. Suppose someone catches some fish and sells the unprocessed fish to me for $100 and then I employ someone to process the fish for $20, and then I sell the fish for $150. How much total value has been added to the economy here?  The answer is less than $150, because the buyer of the finished product would otherwise have just spent his $150 elsewhere (although presumably he would not have derived as much utility from the alternative purchase—thus his decision to spend his money on my fish when the option arose). Equally, the person I hired for $20 would have been hired somewhere else had I not hired him (although presumably for less than $20, otherwise he would not have accepted my job offer).

The upshot of this is that, in your paper, you should avoid adding the primary and secondary values together, or alternatively you should find a good reference to say that adding them is OK (contrary to what I have said above).  It’s possible that I’ve misunderstood what you’ve done (e.g., maybe in what you describe as the “Processing Margin”, you or the references you cite are somehow accounting for the issues I outline above), in which case you just need to explain it a bit better.

Other comments:

•    Figure 1 legend: “Figure and Maxtent potential distribution model”.  Do you mean “MaxEnt”?

•    p6: “the following simple linear model”  Prefer just “the following simple model”, because “linear model” will make most readers think of linear regression models, which is not what you are doing.

•    p9 and throughout: “kgs” -> “kg”

I have read this submission. I believe that I have an appropriate level of expertise to confirm that it is of an acceptable scientific standard, however I have significant reservations, as outlined above.

# Response

**Published:** 2014-02-28

**Authors:** Megan Mach

**Affiliations:** Center for Ocean Solutions, Stanford University, USA

Dear Dr. Chisholm,

Thank you for taking the time to review our article, we believe it has improved because of your review. I have included our responses and notes on the changes made to the manuscript (in normal font) as a result of your comments (denoted in *italics*).

1. *"How are “Processing margin” and “Distribution margin”, which make up the secondary value in Table 2, calculated?"*
  To clarify how processing and distribution margin were calculated we have added specific text to Table 2. This information should not only clarify these two metrics but lay out more clearly how each of the values in the table were calculated. We specifically describe how these were calculated as part of our response to the following comment.
2. **"Does this secondary value represent the difference between the market value and harvested value of the fish?"**
  The processing margin (one of the secondary values) represents the difference between the wholesale market value and the harvest value, however the distribution margin is in addition to wholesale value (15% of processed value, described more below).
  Revisiting the original documentation for these values ( GS Gislason & Associates Ltd., 2007) we caught an error in our processing and distribution values. We had calculated processing margin of shellfish based on the Total fisheries processing margin, however, these values should have been calculated as the difference between Wholesale Value and Landed Value for all shellfish (from both capture and aquaculture, page 12 of GS Gislason & Associates Ltd., 2007). We have corrected the processing margin accordingly for BC in Table 2, from $100m to $71m. This then changes the distribution margin, which is 15% of harvesting + processing margin (the processed value; page 14 of GS Gislason & Associates Ltd., 2007). All relationships are now described as footnotes to Table 2 to ensure the calculations are transparent, including where in the original reference on British Columbia the values originated.
  In addition, the % values in parentheses at the top of each column of Table 2, representing percent value change at increasing crab densities and calories consumed, had been changed but the value in the table had not. This is now corrected. These percentages were calculated from the values in Table 3 as the percent change from the original harvest value to each of the low, medium, and high densities, and low, medium, high calorie diets. Because other values in the table were dependent on these percentages, there was a small shift in the processing margin, labour income, and employment when the percentage was corrected. These value shifts were small and in no way changed the overall message of the table.
3. *"Remove references to a “Total” harvesting, processing and distribution margin, as this total does not consider the possibility of underselling the estimated values."*
  We have removed references to “total” value that include harvesting, processing and distribution from the text as well as from Table 2.
4. *Additional comments*

- *"Figure 1 legend: “Figure and Maxtent potential distribution model”.  Do you mean “MaxEnt”?"*
  Yes, thank you, this has now been corrected.
- *"p6: “the following simple linear model”  Prefer just “the following simple model”, because “linear model” will make most readers think of linear regression models, which is not what you are doing."*
  We have changed the wording to ensure we do not misrepresent our model.
- *"p9 and throughout: “kgs” -> “kg”"*
  This has been corrected throughout the manuscript.
